# Novel gas sensing mechanisms of Pd and Rh-doped h-BN monolayers for detecting dissolved gases (H_2_、CH_4_、and C_2_H_4_) in transformer oil

**DOI:** 10.3389/fchem.2024.1507905

**Published:** 2024-12-06

**Authors:** Jiaming Jiang, Dingqian Yang, Wen Zeng, Zhongchang Wang, Qu Zhou

**Affiliations:** ^1^ College of Engineering and Technology, Southwest University, Chongqing, China; ^2^ State Grid Xinjiang Electric Power Research Institute, Urumqi, Xinjiang, China; ^3^ College of Materials Science and Engineering, Chongqing University, Chongqing, China; ^4^ Department of Quantum and Energy Materials, International Iberian Nanotechnology Laboratory (INL), Braga, Portugal

**Keywords:** Pd-BN, Rh-BN, sensing performance, dissolved gases in transformer oil, DFT

## Abstract

Detecting dissolved gases in transformer oil is crucial for assessing the operational status of transformers. The gas composition in transformer oil can reflect the health status of the equipment and help identify potential failure risks in a timely manner. Based on density functional theory (DFT), Pd and Rh atoms were doped into the h-BN monolayer, and the most stable adsorption structures for each were first explored. Then, the sensing performance of the Pd-doped and Rh-doped h-BN monolayers for H_2_, CH_4_, and C_2_H_4_ gases was analyzed. The results indicate that Pd-BN and Rh-BN exhibit enhanced sensitivity to H_2_ and C_2_H_4_ gases compared to pristine h-BN. However, they show poor adsorption characteristics for CH_4_. Both Pd-BN and Rh-BN demonstrate strong chemisorption for H_2_ and C_2_H_4_. In contrast, CH_4_ adsorption is predominantly physisorbed. The desorption time of H_2_ from Pd-BN at 398 K is 164 s, reflecting its excellent desorption performance. Additionally, Pd-BN and Rh-BN monolayers exhibit exceptional C_2_H_4_ capture capabilities, with adsorption energies of −1.697 eV and −2.188 eV, respectively, indicating their potential as C_2_H_4_ gas adsorbents. These findings provide theoretical insights for selecting materials for dissolved gas detection in oil and lay the groundwork for the development of Pd-BN and Rh-BN-based gas sensors.

## Introduction

Oil-immersed transformers, as a crucial component in the power system, have their operational status directly affecting the stability of the power system (Mu et al., 2022). However, over extended periods of operation, oil-immersed transformers often experience issues such as overheating faults, aging faults, and partial discharge faults (Peng et al., 2023; Long et al., 2024). The insulating oil within transformers can be affected, leading to the generation of fault characteristic gases dissolved in the oil, such as hydrogen (H₂) and various low molecular weight hydrocarbons, including methane (CH₄) and ethylene (C₂H₄) (Zhang et al., 2019; Chen et al., 2022; Li B. L. et al., 2022; Wu et al., 2024). Thermal faults, aging faults, and partial discharge faults not only pose serious threats to the safe operation and service life of transformers but also impact the performance of the insulation, leading to its degradation (Bustamante et al., 2019; Wang et al., 2021; Manoj et al., 2023; Jiang et al., 2024). Therefore, timely detection of these faults in transformers is crucial. As the internal insulation condition of oil-immersed transformers changes and insulation failure intensifies, the types, quantities, and the rates of generation of fault characteristic gases dissolved in the insulation oil also vary accordingly (Du et al., 2018; Zhou et al., 2023). Therefore, analyzing the composition and concentration of fault characteristic gases in the oil aids in assessing the condition of internal insulation faults and predicting the operational status of the transformer (Elele et al., 2022; Ali et al., 2023; Zhong et al., 2023).

Two-dimensional nanomaterials have recently become a focal point in gas sensor research due to their high specific surface area, enhanced chemical reactivity, and excellent carrier mobility. These attributes make them particularly promising for advancing sensor technology (Jiang et al., 2024; Kumar et al., 2024; Long et al., 2024; Zhang et al., 2024). Furthermore, the application of sensors based on two-dimensional nanomaterials for detecting dissolved gases in transformer oil has been extensively studied (Zhou et al., 2018; Li Z. H. et al., 2022; Mishra et al., 2022; Hussein et al., 2023; Gao M. Y. et al., 2024). h-BN is a typical graphene-like material that, compared to conventional graphene, exhibits superior thermal stability and chemical stability (Wang et al., 2017; Ngamprapawat et al., 2023; Ogawa et al., 2023). Pd and Rh have attracted significant attention due to their excellent carrier mobility and outstanding catalytic performance in gas interactions. They are considered suitable dopant metals for enhancing surface properties (Oyo-Ita et al., 2023; Sun et al., 2023; Gao G. et al., 2024; Yao et al., 2024; Yu et al., 2024). Doping transition metal atoms such as Pd and Rh can significantly enhance the material’s reactivity and gas transport capabilities (Kou et al., 2018; Zhao and Wu, 2018; Ni et al., 2020; Peng et al., 2021; Du and Wu, 2022). Based on this, it is anticipated that doping with Pd and Rh atoms could improve the adsorption characteristics of monolayer h-BN towards dissolved characteristic gases in transformer oil, thereby facilitating the detection of these gases. However, research on the gas-sensing mechanisms of Pd and Rh-doped h-BN monolayers in relation to dissolved characteristic gases in oil is still limited.

This study investigates the use of Pd and Rh transition metal atoms doped separately into monolayer h-BN as sensing materials for H₂, CH₄, and C₂H₄. Based on first-principles density functional theory (DFT), the adsorption processes of Rh-doped and Pd-doped h-BN monolayers are calculated and analyzed. DFT calculations have been extensively utilized in previous research within the field of sensing properties. Therefore, this study contributes to the development of gas sensors for detecting dissolved gases in transformer oil. The findings from this research not only advance our understanding of gas adsorption mechanisms but also have significant implications for enhancing the safety and reliability of power systems. By improving the detection of dissolved gases in transformer oil, this study contributes to the development of more effective fault detection technologies, ultimately promoting the stability and efficiency of electrical infrastructures.

### Parameter settings

All theoretical calculations in this study were performed using the DMol3 package within Materials Studio (MS) based on Density Functional Theory (DFT). We employed the Perdew–Burke–Ernzerhof (PBE) functional within the Generalized Gradient Approximation (GGA) to describe exchange-correlation potentials (Wang et al., 2024). To more accurately capture long-range interactions and van der Waals forces, the semi-empirical dispersion correction method (DFT-D) proposed by Grimme was used to account for van der Waals interactions (Grimme, 2006). The calculations utilized a Double Numerical with Polarization (DNP) atomic orbital basis set to ensure high computational quality and employed DFT semi-core pseudopotentials (DSPP) for Pd and Rh atoms to handle relativistic effects (Zeng et al., 2020). For geometry optimization, a 3 × 3 × 1 Monkhorst–Pack grid was used for k-point sampling in the Brillouin zone. The convergence criteria were set as: maximum force, maximum displacement, and energy tolerance of 0.002 Ha/Å, 0.005 Å, and 10^−5^ Ha, respectively. The self-consistent field (SCF) convergence threshold for static electronic structure calculations was set to 10^−6^ Ha, with a Gaussian broadening Sigma parameter of 0.005 Ha to ensure accuracy in total energy results. The smearing value for thermal broadening effects is set to 0.005 Ha. A 4 × 4 × 1 supercell of the h-BN monolayer was constructed, with a 15 Å vacuum region to avoid interactions between adjacent supercells. The optimized lattice constant of the h-BN monolayer was 2.5 Å, consistent with other theoretical values.

To determine the most stable doping structure, we employed the binding energy equation. Formula 1 can be used to calculate the binding energies ( $E_{b}$ ) of Rh and Pd atoms at each site of the h-BN monolayer (Chen et al., 2024).

$$E_{b}=E_{ATOM-BN}-E_{ATOM}-E_{BN}$$ (1)

Where $E_{ATOM-BN}$ 、 $E_{ATOM}$ and $E_{BN}$ represent the total energy of the Rh or Pd doped complex, the Rh or Pd atom, and the undoped BN, respectively. Typically, a negative binding energy $E_{b}$ indicates a spontaneous exothermic reaction.

The adsorption energy $E_{ads}$ can reflects the energy change of the entire adsorption system, allowing for the assessment of the strength of the adsorption reaction, the specific expression is shown in Formula 2 (Yu et al., 2024):

$$E_{ads}=E_{gas-sub}-E_{sub}-E_{gas}$$ (2)

Where $E_{gas-sub}$ , $E_{sub}$ and $E_{gas}$ stands for the total energy of the adsorption system, the energy of gas-sensitive material and gas molecule, respectively. When $E_{ads}$ < 0, it indicates that the adsorption reaction is exothermic and chemical, making it spontaneous. If the absolute value of the $E_{ads}$ > 0.8 eV, it can be considered strong chemical adsorption. Conversely, when $E_{ads}$ > 0, it indicates that the adsorption reaction is endothermic and physical, and thus not spontaneous.

The amount of charge transfer can reflect the change in the charge carried by gas molecules before and after the adsorption reaction, thus allowing the assessment of the impact of the adsorption reaction on the electrical conductivity of gas-sensitive materials. This can be expressed by Formula 3 (Tang et al., 2020):

$$Q_{t}=Q_{a}-Q_{b}$$ (3)

Where $Q_{a}$ and $Q_{b}$ denote the amount of charge carried by gas molecules after adsorption and the amount of charge carried before adsorption.

## Results and discussion

### Establishment of the doping model

To determine the optimal doping structure of Pd and Rh atoms on a single layer of h-BN, we considered doping at four distinct sites: atop N atoms (TN), atop B atoms (TB), at the midpoint of B-N bonds (TM), and at the center of the BN hexagons (TH). By comparing the binding energies ( $E_{b}$ ) of each doping configuration at these sites, we found that the most stable doping structure for both Pd and Rh atoms is at the TN site, where both exhibit the lowest binding energy and adsorption distance.

**FIGURE 1:**
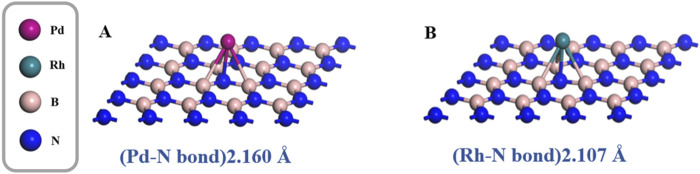
Geometric structure of **(A)** Pd-BN, **(B)** Rh-BN.

**FIGURE 2:**
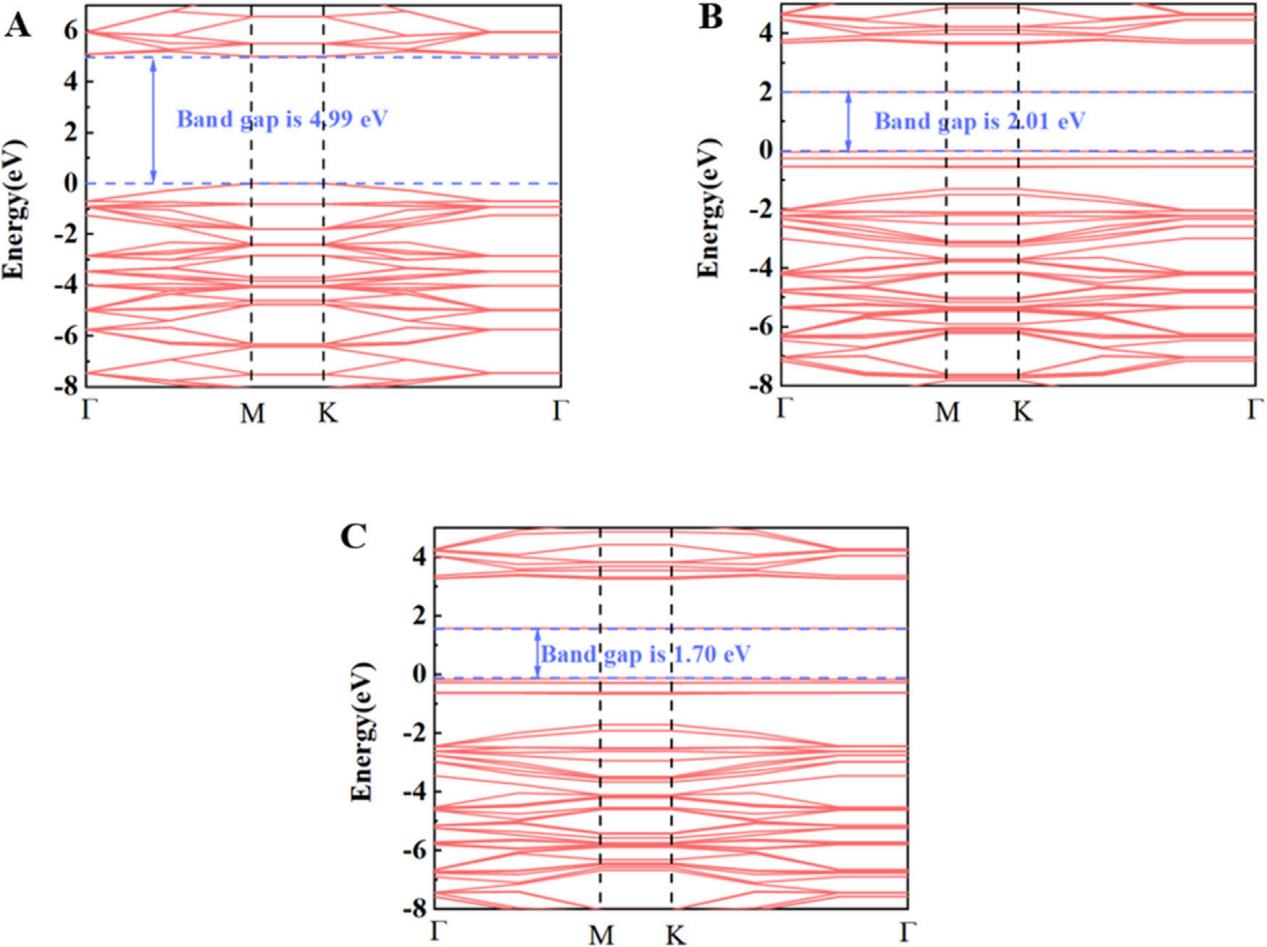
Band structure of **(A)** h-BN, **(B)** Pd-BN, **(C)** Rh-BN.

The optimized geometric models of Pd-doped and Rh-doped h-BN monolayers are shown in Figure 1. The binding energies for the two structures are −1.123 eV and −1.396 eV, respectively, with Pd and Rh atoms located at distances of 2.160 Å and 2.107 Å from the N atoms. Additionally, compared to the intrinsic band gap of h-BN (4.988 eV) (Guo et al., 2022), the band gaps of Pd-doped BN and Rh-doped BN are significantly reduced to 2.055 eV and 1.697 eV, respectively. The band gap diagrams are presented in Figure 2. These results indicate that both Pd and Rh atoms significantly enhance the electrical conductivity of the monolayer, demonstrating effective doping.

**FIGURE 3:**
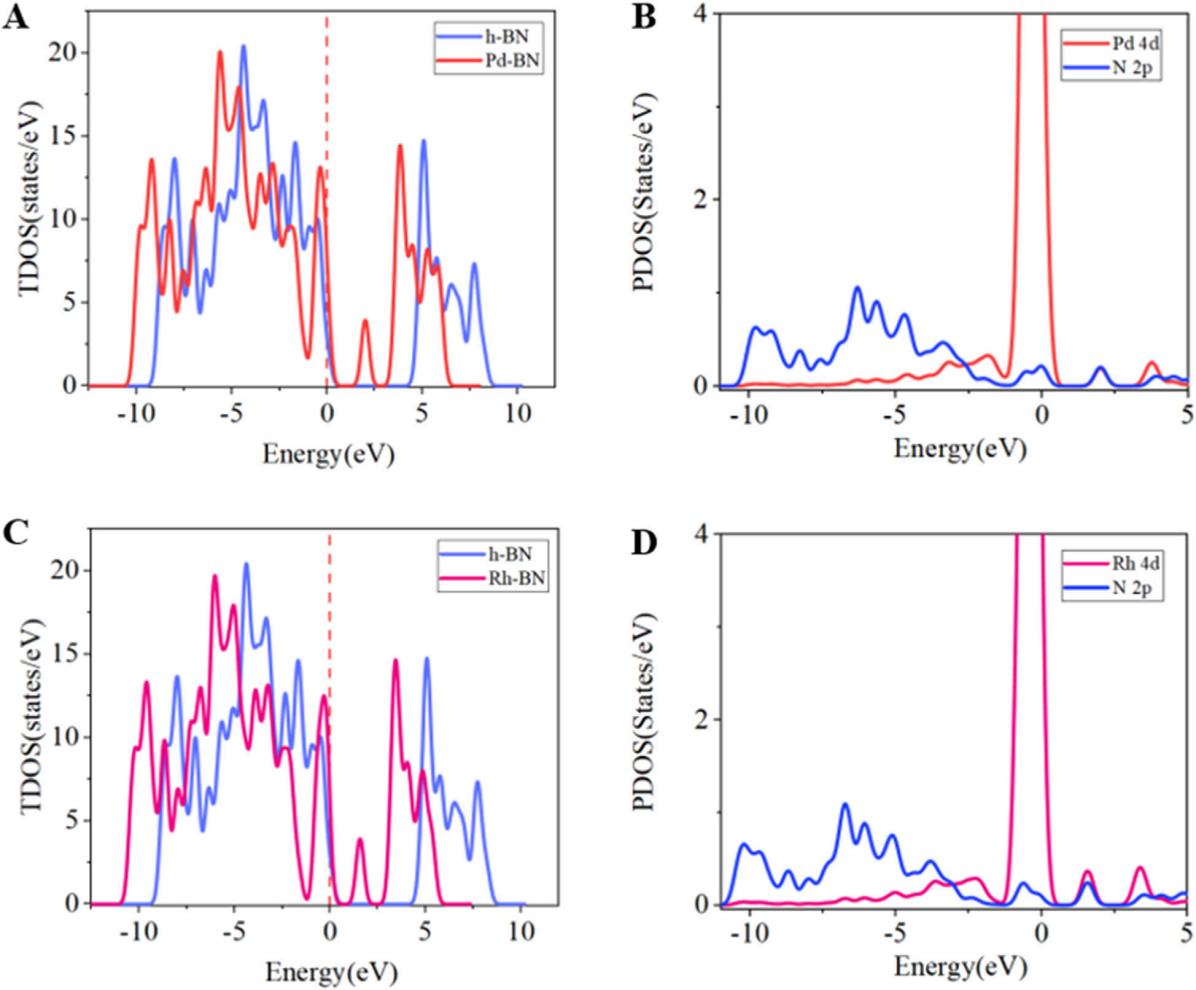
**(A)** TDOS of h-BN and Pd-BN, **(B)** PDOS of Pd-BN, **(C)** TDOS of h-BN and Rh-BN, **(D)** PDOS of Rh-BN.

To further analyze the electronic behavior of Pd-BN monolayers, Rh-BN monolayers, and pure h-BN monolayers, we performed total density of states (TDOS) analysis for all three systems. The results indicate a significant contribution of Pd and Rh atoms to the TDOS. In both Pd-BN and Rh-BN systems, new peaks appear to the right of the Fermi level, suggesting that electron transfer from the valence band to the conduction band has become easier, consistent with the observed reduction in band gap. Additionally, data presented in Figure 3B reveal substantial overlap between Pd 4d and N 2p orbitals within the range of −10.5 eV–5 eV, highlighting strong hybridization between Pd atoms and the BN layer. Similarly, Figure 3D shows notable overlap between Rh 4d and N 2p orbitals, indicating a strong bonding interaction between Rh atoms and h-BN. These analyses underscore the impact of Pd and Rh atoms on the electronic behavior of the BN layer and their significant modification of the material’s electronic structure.

### Adsorption properties

To investigate the sensing and adsorption properties of monolayers of Pd-BN and Rh-BN for H₂, CH₄, and C₂H₄ gases, adsorption models for different adsorption behaviors were established. Optimization calculations yielded the optimal adsorption configurations for these gases on both monolayers. The adsorption energies, charge transfers, adsorption distances, and band gaps for the six adsorption systems are summarized in Table 1.

**TABLE 1:** Adsorption parameters of the Pd-BN monolayer and Rh-BN monolayer to dissolved gases in transformer oil.

Gas	Model	$E_{\text{ads}}$ (eV)	$Q_{t}\left(e\right)$	D(Å)	Bandgap (eV)
H_2_	Pd-BN	−1.123	−0.044	1.736	3.421
	Rh-BN	−1.516	−0.027	1.686	2.944
CH_4_	Pd-BN	−0.121	−0.028	1.968	3.703
	Rh-BN	−0.116	0.028	1.886	3.364
C_2_H_4_	Pd-BN	−1.697	−0.100	2.157	2.869
	Rh-BN	−2.188	−0.090	2.120	2.601

**FIGURE 4:**
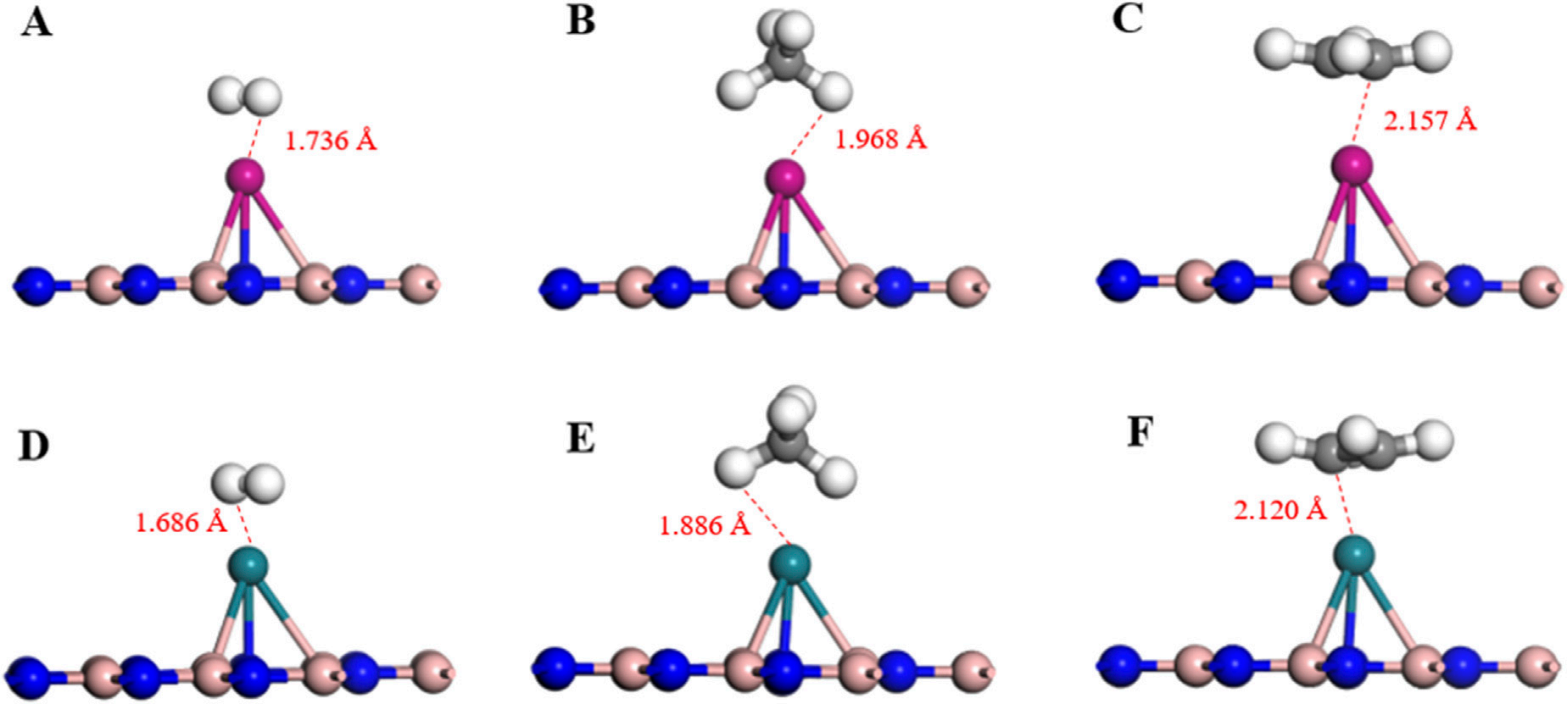
The optimal adsorption configuration of **(A)** Pd-BN/H_2_, **(B)** Pd-BN/CH_4_, **(C)** Pd-BN/C_2_H_4_, **(D)** Rh-BN/H_2_, **(E)** Rh-BN/CH_4_, **(F)** Rh-BN/C_2_H_4_.

Based on the adsorption parameters presented in Table 1, it can be preliminarily concluded that the Pd-BN monolayer exhibits favorable adsorption properties for H₂ and C₂H₄ molecules, with adsorption energies of −1.123 eV and −1.697 eV, respectively. In contrast, the adsorption of CH₄ molecules is less effective, as evidenced by its relatively low adsorption energy of −0.121 eV. H_2_ and C_2_H_4_ have relatively short adsorption distances on the Pd-BN monolayer, as shown in Figure 4, measuring 1.736 Å and 2.157 Å, respectively. This indicates a strong interaction between H₂, C₂H₄ molecules and the Pd-BN monolayer. The negative adsorption energies suggest that these adsorption processes are spontaneous, with considerable adsorption energy magnitudes. The larger absolute values of the adsorption energies for H₂ and C₂H₄ imply that the adsorption of these molecules on the Pd-BN monolayer is likely chemisorptive. Conversely, the negligible charge transfer for CH₄ suggests that its interaction with the monolayer is primarily a weak physisorption.

Based on the adsorption parameters for H₂, CH₄, and C₂H₄ molecules on the Rh-BN Based on the adsorption parameters of H₂ and C₂H₄ on the Rh-BN monolayer, it is similarly evident that there is a strong interaction between the Rh-BN monolayer and these two gas molecules. The adsorption energy of H₂ on the Rh-BN monolayer is −1.516 eV, and the adsorption distance, as shown in Figure 4D, is 1.686 Å. Indicating a significant interaction between them. Additionally, the Hirshfeld charge distribution method reveals that H₂, acting as an electron donor, contributes 0.027 e to the Rh-BN monolayer, suggesting a notable charge transfer from the H₂ molecule to the Rh-BN monolayer. The adsorption energy and charge transfer for CH₄ are minimal, indicating that CH₄ interacts with the Rh-BN monolayer through weak physisorption. Conversely, the adsorption energy and charge transfer for C₂H₄ are substantial, reflected by the larger adsorption energy and significant charge transfer, suggesting strong chemisorption of C₂H₄ on the Rh-BN monolayer.

### Electronic behavior of gas adsorption structures on Pd-BN and Rh-BN based on DOS analysis

**FIGURE 5:**
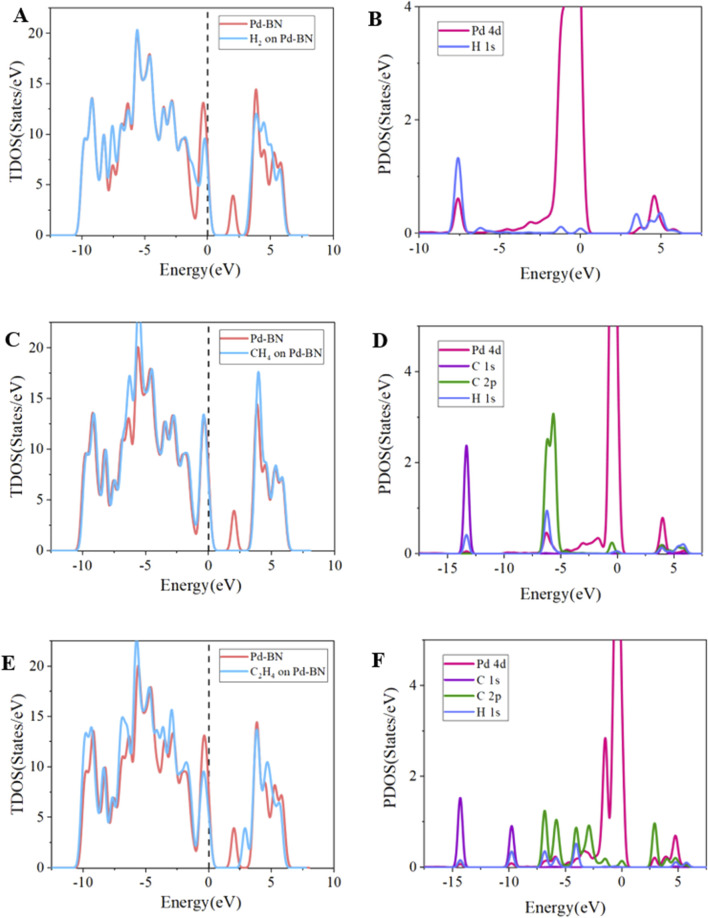
Tdos of **(A)** Pd-BN/H_2_, **(B)** Pd-BN/CH_4_, **(C)** Pd-BN/C_2_H_4_, PDOS of **(D)** Pd-BN/H_2_, **(E)** Pd-BN/CH_4_, **(F)** Pd-BN/C_2_H_4_.

To further elucidate the gas-sensing mechanisms and electronic behavior of Pd-BN and Rh-BN monolayers with respect to H_2_, CH_4_, and C_2_H_4_ molecules, we analyzed the total density of states (TDOS) and partial density of states (PDOS) of Pd-BN and Rh-BN monolayers under the adsorption of these three gases, as shown in Figure 5.

Firstly, focusing on the TDOS distribution for Pd-BN monolayer in each adsorption system, the red curve represents the TDOS distribution of the bare Pd-BN monolayer, while the blue curve denotes the TDOS distribution after gas adsorption. Significant differences are evident in the TDOS plots for H_2_, CH_4_, and C_2_H_4_, both before and after adsorption. Notably, the TDOS near the Fermi level shows substantial changes in the H_2_ and CH_4_ adsorption systems. The density of states peak of Pd-BN in the range of 1.5 eV–3 eV disappears, making it harder for electrons to transfer from the valence band to the conduction band, consistent with the increase in bandgap observed in Figure 2B. The adsorption of H_2_ and CH_4_ reduces the electrical conductivity of the Pd-BN monolayer. In the C_2_H_4_ adsorption system, a shift in the density of states peak to the right of the Fermi level is observed, which also contributes to a certain increase in the bandgap.

In the PDOS curves for the three gas adsorption systems on Pd-BN, overlaps among curves representing different atomic orbitals indicate orbital hybridization. In the H_2_ adsorption system shown in Figure 5B, strong hybridization between H 1s and Pd 4d peaks is observed in the ranges of −7.5 eV and 3 eV–6 eV, which corroborates the high adsorption energy of H_2_. For the CH_4_ adsorption system, despite multiple overlaps between Pd 4d and C 2p, H 1s orbitals, the smaller peak value of Pd 4d indicates less effective adsorption, with physical adsorption being predominant, consistent with the adsorption energy analysis. In the C_2_H_4_ adsorption system, extensive overlap is observed between −15 eV and −2.5 eV, and hybridization occurs between 2.5 eV and 5 eV, indicating strong orbital hybridization between Pd 4d, C 2p, and H 1s orbitals, and strong Pd-C bonding. Additionally, these overlaps significantly deform the TDOS, explaining the shift observed in the TDOS curves. The overlap between Pd 4d and C 2p orbitals in the C_2_H_4_ adsorption system is greater than that in the H_2_ and CH_4_ systems, verifying the stronger chemical interaction in the C_2_H_4_ adsorption system.

**FIGURE 6:**
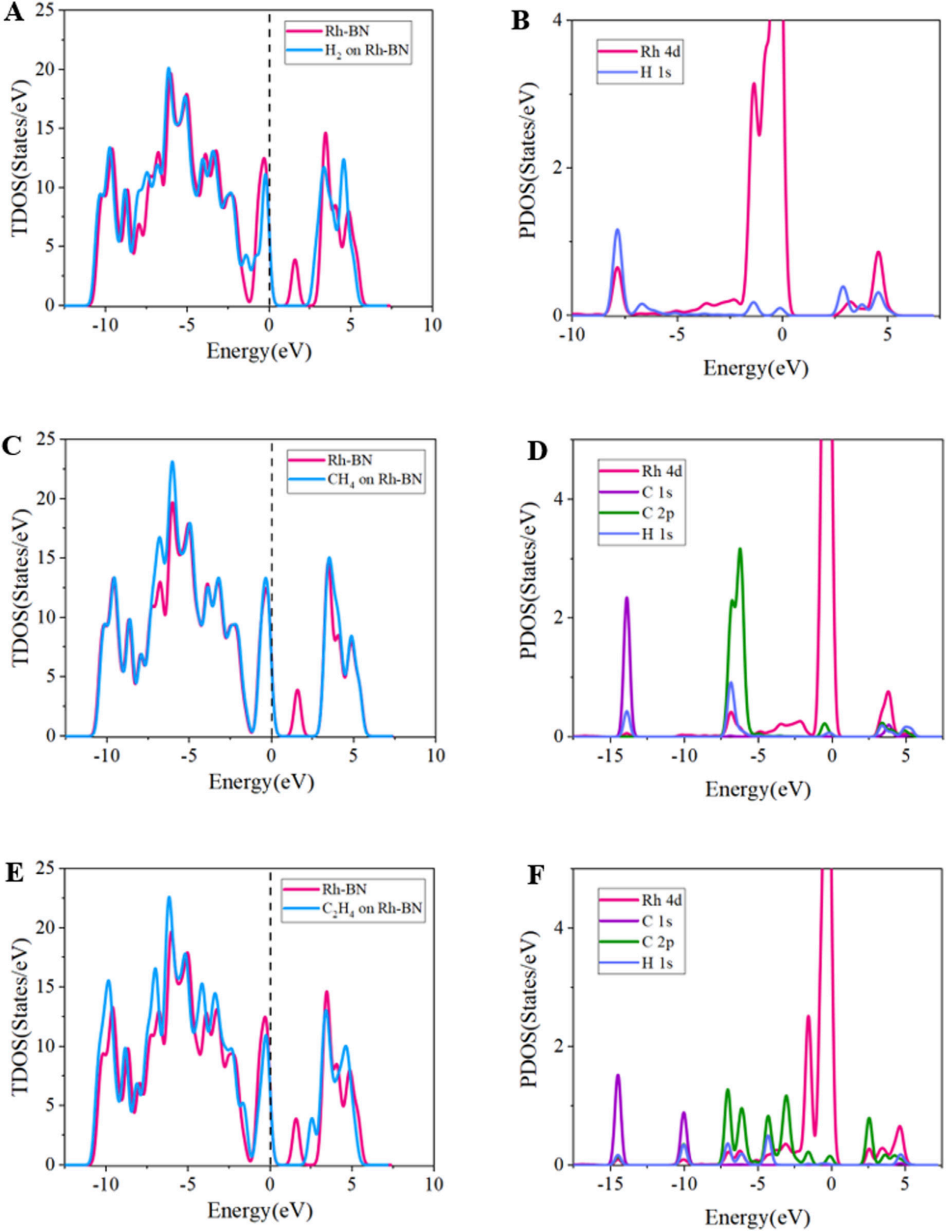
Tdos of **(A)** Rh-BN/H_2_, **(B)** Rh-BN/CH_4_, **(C)** Rh-BN/C_2_H_4_, PDOS of **(D)** Rh-BN/H_2_, **(E)** Rh-BN/CH_4_, **(F)** Rh-BN/C_2_H_4_.

The TDOS and PDOS analysis for the Rh-BN monolayer under adsorption of H_2_, CH_4_, and C_2_H_4_ reveals significant changes in electronic structure. As shown in Figure 6, in the H_2_ adsorption system, the TDOS shows that the density of states peak on the right side of the Fermi level in the 1 eV–2 eV range disappears, indicating that electron transfer from the valence band to the conduction band becomes more difficult. This observation aligns with the observed increase in bandgap in the adsorption system. For the CH_4_ adsorption system, a similar disappearance of the density of states peak on the right side of the Fermi level occurs, but with considerable curve overlap across a broad range, suggesting that CH_4_ adsorption has a minimal impact on the electronic density distribution of Rh-BN. In the C_2_H_4_ adsorption system, the TDOS shows a notable shift of the density of states peak to higher energy levels compared to the pristine Rh-BN, with an increased intercept width at the fermi level. This implies that C_2_H_4_ adsorption enlarges the system’s bandgap, leading to a downward shift of the Fermi level and decreased electrical conductivity of the Rh-BN monolayer.

The PDOS for Rh-BN/H_2_ shows significant overlap between Rh 4d and H 1s orbitals at −8 eV, along with hybridization between 2.5 eV and 5 eV, indicating a strong binding interaction between Rh-BN and H_2_. This explains the high adsorption energy and structural stability of the Rh-BN/H_2_ system. For Rh-BN/CH_4_, the PDOS results show limited orbital overlap between Rh 4d and C 2p, H 1s orbitals at −7.5 eV, reflecting poor adsorption efficacy of CH_4_. In the Rh-BN/C_2_H_4_ system, the PDOS reveals extensive and continuous overlap between Rh 4d and C 2p, H 1s orbitals in the −7.5 eV to −2.5 eV range, signifying strong chemical adsorption of C_2_H_4_ on the Rh-BN monolayer, consistent with the high $E_{ad}$ value and the stable geometric configuration of the C_2_H_4_ adsorption system.

### Band gap study based on frontier orbital theory

It is generally accepted that the electrical conductivity is related to the band gap through the Formula 4:

$$\sigma \propto \exp\left(\frac{-E_{g}}{k_{B}T}\right)$$ (4)

Where $k_{B}$ is the Boltzmann constant, T is the temperature.

**FIGURE 7:**
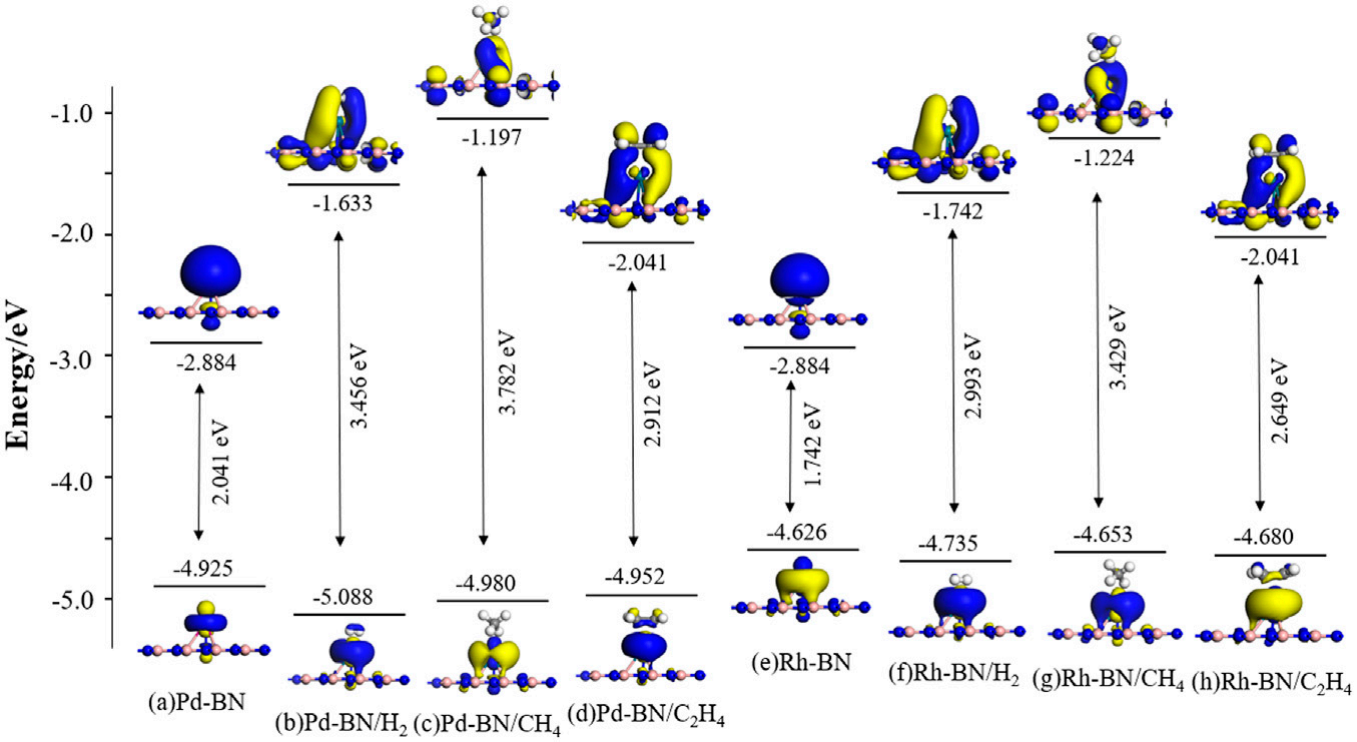
Distribution of HOMO and LUMO in Monolayer Pd-BN and Rh-BN and their adsorption systems with three gas molecules.

From the above equation, it can be seen that the electrical conductivity is inversely proportional to the band gap under certain temperature conditions. In this section, frontier orbital theory is employed to quantitatively assess changes in the band gap by calculating the energies of the highest occupied molecular orbital (HOMO) and the lowest unoccupied molecular orbital (LUMO). This allows for an analysis of how adsorption behavior impacts the electrical conductivity of the material. The HOMO and LUMO distributions for the Pd-BN monolayer, Rh-BN monolayer, and the adsorption systems of three gases on different monolayers are shown in Figure 7.

From Figure 7, it can be observed that before adsorption the HOMO and LUMO of the Pd-BN and Rh-BN systems are primarily localized around the dopant atoms. After gas adsorption, the distributions of HOMO and LUMO shift significantly. The HOMO becomes predominantly localized near the gas molecules, while the LUMO begins to interact with the BN monolayer. This change is likely due to electron redistribution induced by gas adsorption.

The bandgap values for each system can be calculated based on the energies of the HOMO and LUMO. Consistent with the conclusions from the density of states analysis, the bandgaps of the systems with adsorbed H_2_, CH_4_, and C_2_H_4_ gases are increased compared to their non-adsorbed counterparts. The effect of CH_4_ adsorption on the bandgap of Pd-BN and Rh-BN monolayers is less pronounced, which aligns with the results obtained from the density of states analysis.

### Response recovery characteristics

For gas sensors, the desorption time is a crucial parameter for assessing the repeatability of the sensor. According to the Van’t Hoff-Arrhenius expression, the desorption time can be calculated using the following Formula 5.

$$\tau =A^{-1}{\mathrm{e}}^{-E_{\alpha }/\left(k_{B}T\right)}$$ (5)

**FIGURE 8:**
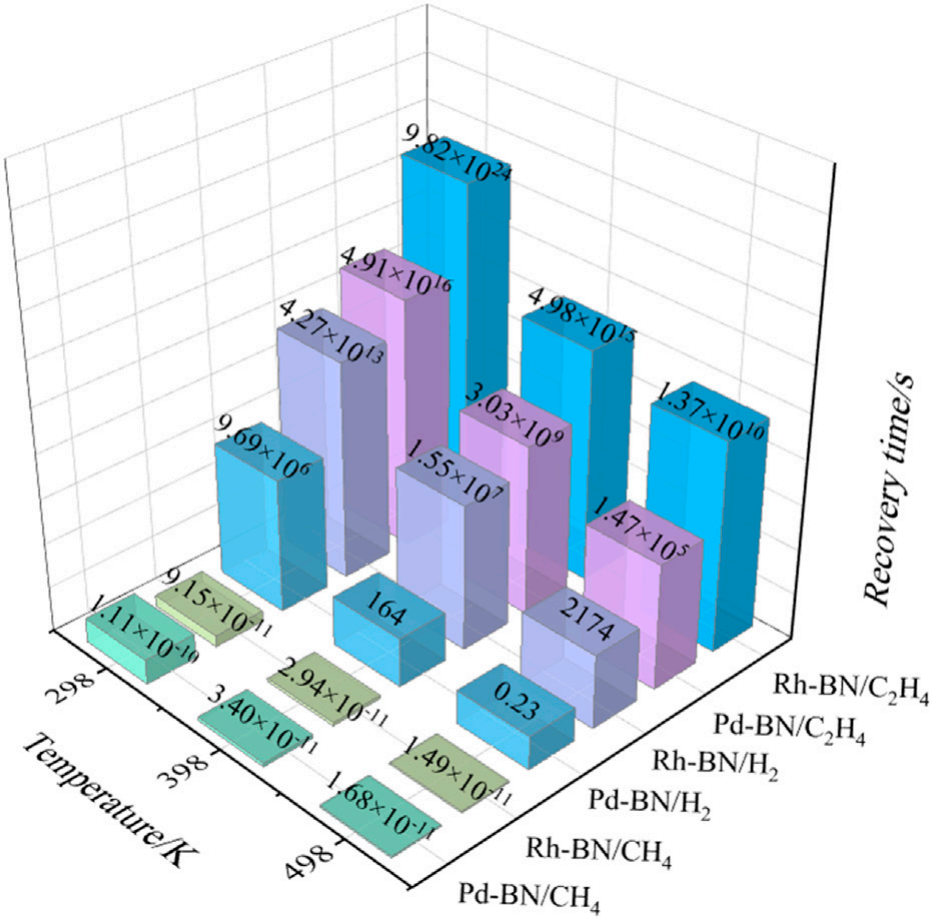
Desorption time of three gas molecules from the Pd-BN monolayer and Rh-BN monolayer.

In the formula, A represents the attempt frequency ( $A=10^{12}\cdot s^{-1}$ ), T is the temperature; $k_{B}$ represents the Boltzmann constant ( $k_{B}=8.62\times 10^{-5}\text{eV}/K$ ); $E_{\alpha }$ is the activation energy required for desorption, assumed to be the same as $E_{ads}$ . The desorption times of H₂, CH₄, and C₂H₄ at temperatures of 298 K, 398 K, and 498 K from the surfaces of Pd-BN and Rh-BN monolayers are illustrated in Figure 8.

As illustrated in Figure 8, the desorption times for CH₄ molecules from both Pd-BN and Rh-BN monolayers are remarkably brief, with corresponding adsorption energies of −0.121 eV and −0.116 eV, respectively. These short desorption times suggest that CH₄ exhibits a very low likelihood of being effectively adsorbed by either the Pd-BN or Rh-BN monolayers, rendering it unsuitable as a target gas for sensors utilizing these materials. In contrast, H₂ molecules demonstrate more favorable desorption characteristics from the Pd-BN monolayer, requiring only 164 s at 398 K. This indicates that the Pd-BN monolayer is particularly well-suited for the development of sensitive materials for H₂ gas detection.

Conversely, for C₂H₄, even at elevated temperatures of 498 K, the desorption times from both Pd-BN and Rh-BN monolayers remain inadequate. Given that C₂H₄ is a known carcinogen and poses risks to both atmospheric quality and water sources, there is potential for the Pd-BN and Rh-BN monolayers to be utilized as effective adsorbents for the remediation of C₂H₄ gas.

### Comparison of gas adsorption characteristics of h-BN with Pd-BN and Rh-BN monolayers

This study compares the adsorption performance of dissolved characteristic gases in oil on Pd-BN monolayers, Rh-BN monolayers, and native h-BN. It further investigates the potential of h-BN monolayers as gas-sensing materials in the power industry.

The comparison results are presented in Table 2. The intrinsic h-BN exhibits suboptimal adsorption characteristics for dissolved target gases such as H₂, CH₄, and C₂H₄ in oil compared to the doped Pd-BN and Rh-BN monolayers. Both the absolute values of the adsorption energies and charge transfer amounts are relatively low, with larger adsorption distances, indicating that intrinsic h-BN is not sensitive to H₂, CH₄, and C₂H₄.

**TABLE 2:** Comparison of adsorption characteristics of h-BN with Pd-BN and Rh-BN monolayers.

Adsorption system	Gas	$E_{\text{ads}}$ (eV)	$Q_{t}$ (e)	D(Å)
**Pd-BN**	H_2_	−1.123	−0.044	1.736
	CH_4_	−0.121	−0.028	1.968
	C_2_H_4_	−1.697	−0.100	2.157
**Rh-BN**	H_2_	−1.516	−0.027	1.686
	CH_4_	−0.116	0.028	1.886
	C_2_H_4_	−2.188	−0.090	2.120
**h-BN**	H_2_	−0.070	−0.004	3.014
	CH_4_	−0.072	−0.013	3.038
	C_2_H_4_	−0.102	−0.007	3.320

In contrast, the adsorption energies for the various gas systems are significantly increased in both Pd-BN and Rh-BN compared to intrinsic h-BN, particularly for H₂ and C₂H₄, which show stronger chemisorption effects. Both doped monolayers exhibit a strong capture capability for C₂H₄, with a tendency for reduced desorption. Among the doped variants, Pd-BN demonstrates a superior desorption effect for H₂ compared to Pd-BN, with a recovery time of 164 s at 398 K, making Pd-BN more favorable as a hydrogen-sensitive material. Doping with Pd or Rh atoms effectively enhances the gas-sensing performance of h-BN monolayers for dissolved target gases in oil, proving to be an effective modification strategy.

## Conclusion

This study constructed models of Pd-doped and Rh-doped h-BN monolayers to analyze their adsorption characteristics for H₂, CH₄, and C₂H₄ gases found in transformer oil. The findings demonstrate that both Pd-BN and Rh-BN monolayers exhibit strong chemical adsorption for H₂ and C₂H₄, characterized by high adsorption energies, short distances, and significant charge transfer, while their interaction with CH₄ is primarily governed by van der Waals forces. Notably, Pd-BN and Rh-BN monolayers showed reduced electrical conductivity after gas adsorption, highlighting their suitability for gas detection; the Pd-BN monolayer, in particular, has a desorption time of only 164 s for H₂ at 398 K, indicating its potential in hydrogen sensing applications. Furthermore, the incorporation of Pd and Rh enhances the adsorption energy and charge transfer capabilities compared to intrinsic h-BN, positioning these materials as promising candidates for dissolved gas sensors in transformer oils and providing a solid theoretical basis for future development.

## Data Availability

The original contributions presented in the study are included in the article/supplementary material, further inquiries can be directed to the corresponding author.
